# Time-resolved synchrotron X-ray micro-tomography datasets of drainage and imbibition in carbonate rocks

**DOI:** 10.1038/sdata.2018.265

**Published:** 2018-12-11

**Authors:** Kamaljit Singh, Hannah Menke, Matthew Andrew, Christoph Rau, Branko Bijeljic, Martin J. Blunt

**Affiliations:** 1 Qatar Carbonates and Carbon Storage Research Centre, Department of Earth Science and Engineering, Imperial College London, SW7 2AZ London, U.K; 2 Carl Zeiss X-ray Microscopy Inc., Pleasanton, CA, U.S.A; 3 Diamond Light Source, Harwell Science and Innovation Campus, Didcot, U.K

**Keywords:** Environmental sciences, Imaging techniques, Fluid dynamics, Engineering, Carbon capture and storage

## Abstract

Multiphase flow in permeable media is a complex pore-scale phenomenon, which is important in many natural and industrial processes. To understand the pore-scale dynamics of multiphase flow, we acquired time-series synchrotron X-ray micro-tomographic data at a voxel-resolution of 3.28 μm and time-resolution of 38 s during drainage and imbibition in a carbonate rock, under a capillary-dominated flow regime at elevated pressure. The time-series data library contains 496 tomographic images (gray-scale and segmented) for the complete drainage process, and 416 tomographic images (gray-scale and segmented) for the complete imbibition process. These datasets have been uploaded on the publicly accessible British Geological Survey repository, with the objective that the time-series information can be used by other groups to validate pore-scale displacement models such as direct simulations, pore-network and neural network models, as well as to investigate flow mechanisms related to the displacement and trapping of the non-wetting phase in the pore space. These datasets can also be used for improving segmentation algorithms for tomographic data with limited projections.

## Background & Summary

Understanding the dynamics of multiphase fluid flow in permeable media is important in many processes such as water infiltration in soils, oil recovery from reservoir rocks, geo-sequestration of supercritical CO_2_ to address global climate change, and subsurface non-aqueous phase liquid contaminant transport^1–6^. These processes have been studied for decades in two dimensions using micro-models and microfluidic devices^7–11^, which do not provide a complete picture of the dynamics of multiphase flow in a complex realistic permeable medium. In three dimensions, previous studies have used lab-based X-ray micro-tomography to visualize immiscible fluids in porous media at end states^12–16^, i.e., before and after fluid injection, which provide detailed information on the static distribution of fluids but do not describe the dynamics of their transport mechanisms. Time-resolved imaging in lab-based instruments is restricted due to a low photon flux, which results in a time resolution of the order of hours. To obtain a higher photon flux, several studies have used synchrotron X-ray imaging to investigate drainage (displacement of a wetting fluid by a non-wetting fluid) and imbibition (displacement of a non-wetting by a wetting fluid) processes in glass beads and natural porous media (carbonates and sandstones)^4,5,17–24^.

We conducted a high-resolution fast synchrotron X-ray micro-tomographic imaging study of the displacement of immiscible fluids during drainage and imbibition in a carbonate rock sample under a capillary-controlled flow regime at elevated pressure conditions^22^. The dynamic flow displacement experiments were conducted in a 3.8 mm diameter and 10 mm long Ketton limestone sample. The sample was first saturated with brine. The system was then pressurized to 10 MPa, followed by injection of oil (a drainage process for water-wet porous media) from the top of the sample by establishing a pressure gradient of 50 kPa across the sample. When there was no longer any visible change in the oil and brine saturation, the flow was reversed by raising the pressure in the brine pump and establishing a pressure gradient of 22 kPa to start brine injection (an imbibition process for a water-wet porous medium) from the base of the sample. The sample was imaged continuously during drainage and imbibition, with a voxel size of 3.28 μm and acquisition speed of 38 s for each image.

A complete three-dimensional time-resolved image sequence of drainage and imbibition is available online (Data Citation 1 and Data Citation 2). The residual oil at the end of imbibition contains a number of disconnected oil ganglia, these images are also available online (Data Citation 3). These datasets were collected with the aim of investigating pore-scale processes during immiscible fluid displacement under a capillary-dominated flow regime^22^. The time-dependent information can be used to validate models of pore-scale displacement, such as direct simulations^25–27^, pore-network^27,28^ and neural network models^29–31^. Furthermore, the data can be used to quantify how the balance of viscous and capillary forces control the exact nature of trapping, and to further analyze the complex pore-scale processes during immiscible fluid flow in permeable media. In addition, these datasets can be used for improving segmentation algorithms for tomographic data with limited projections.

The time-resolved synchrotron X-ray micro-tomographic images are hosted at the British Geological Survey (BGS) repository, the details of which are provided in the Data Records and Data Citations sections.

## Methods

In this section, we first provide the details of our experimental protocol, followed by synchrotron imaging and image processing. These descriptions overlap with the experimental details provided in Singh, *et al.*^22^.

### Experimental methodology

The experiments were conducted in a Hassler-type flow cell made of carbon fiber that is nearly transparent to X-rays^14^. A 3.8 mm diameter and 10 mm long cylindrical sample of the Ketton limestone rock (from the Ketton quarry, Rutland, UK, which contains >99% calcite with the remaining fraction being quartz^14,32^), which was cleaned with methanol using a Soxhlet extraction apparatus for 24 h followed by drying in a vacuum oven at 100 °C for 24 h, was placed on the top of a low-permeability water-wet Aluminum-silicate ceramic porous plate (3.8 mm diameter and ~ 4 mm long) with a breakthrough pressure of 1.5 MPa (Weatherford Laboratories, Stavanger, Norway) in a Viton sleeve. The Viton sleeve was attached to a metal end piece at the base, which was connected to a high-pressure syringe pump (ISCO-100D, TELEDYNE, U.S.A.) with PEEK tubing (Kinesis, U.K.). A PEEK spacer, with an outer and inner diameter of 4 mm and 2 mm respectively, was placed on the top of the sample, which allowed monitoring of the brine-oil interface while the system was pressurized and prior to oil injection in the water saturated sample. The rock was imaged before starting oil and brine injection (hereafter called the dry reference scan).

The flow loop of the experimental apparatus is shown in Singh, *et al.*^22^. After acquiring a dry reference scan, CO_2_ was injected in the sample to displace air. The brine (1.8 M solution of potassium iodide, puriss, 99.5%, Sigma-Aldrich, U.K.) was injected through the sample from the base at 0.1-0.2 mL/min. A total of 80-100 pore volumes of brine was injected to remove both gaseous and dissolved CO_2_ in brine, ensuring 100% brine saturation. This concentration of the salt solution was pre-selected to obtain an effective X-ray contrast between brine and oil.

The oil (decane, ReagentPlus, ≥ 99%, Sigma-Aldrich, U.K.) was filtered four times through a column of aluminum oxide powder to remove surface active impurities and to obtain a stable brine-oil interfacial tension^33^. The oil was then loaded into the pump and pushed through the flow lines to the upper metal end piece. The metal end piece was then carefully attached to the upper part of the Viton sleeve (on the top of the brine-filled PEEK spacer), avoiding air entrapment during assembly. In this way, the brine-oil interface was established near the top of the PEEK spacer. The core holder was then mounted on the rotation stage of the beamline. The sample was aligned to the X-ray beam. The pressure in the brine, oil and the confining fluid (deionized water) was then raised step-wise to 10 MPa and 11.2 MPa respectively. A higher confining pressure was used to confine the Viton sleeve in which the sample was placed, to avoid any fluid bypassing along the walls of the sample. The brine-oil interface in the PEEK spacer was carefully monitored during pressurizing the fluids. All the pumps were previously calibrated and tested for pressure difference by interconnecting and pressuring them. All the experiments were conducted at ambient temperature (20 °C).

For oil flooding, the pressure of the oil pump was raised by 50 kPa, which resulted in the migration of water-oil interface from the top of the sample. The water-wet porous plate prevented the non-wetting phase (oil) to pass through at the pressure drop used in this experiment, therefore, removing the dead volume during brine flooding as the oil-brine interface was established just at the base of the core sample after drainage. The sample was continuously imaged during drainage. When there was no visible change in the oil and brine phases (found by subtracting the projection data of consecutive tomographic images), the flow was reversed by raising the pressure in the brine pump and establishing a pressure gradient of 22 kPa to start brine injection from the base of the sample. The low-permeability porous plate at the base of the core sample provided low flow rates, which was important to acquire distortion free time-resolved tomographic images. A flow rate of 44.75 nL/min was achieved during imbibition, leading to a Darcy velocity of 3.94 μm/min and a capillary number (*N*_*c*_ = *νμ*/*γ*, where *ν* is the Darcy velocity of the invading fluid, *μ* is the viscosity of the invading fluid, and *γ* is the brine-oil interfacial tension) of 1.26 × 10^−9^, using an interfacial tension of 52.33 ± 0.04 mN/m^34^), representing a capillary-flow regime. The sample was continuously imaged during imbibition.

### Synchrotron imaging and image processing

The time-resolved X-ray micro-tomography was performed at the Diamond Light Source (UK), Beamline I13, using a pink beam with energies in the range of 6 to 30 keV. The low energy X-rays were filtered by placing a set of 0.2 mm pyrolitic graphite, 2.2 mm aluminum, and 0.1 mm gold filters in the beam. This reduced the relative intensity of low-energy X-ray in the polychromatic X-ray spectrum, controlling sample heating. The sample was rotated by 180° during the acquisition of the projection data. It was then brought back to the initial position for the next tomographic image. The X-rays that passed through the sample were converted to visible light by a 250 μm thick CdWO_4_ scintillator; which was then recorded by a PCO Edge camera with 2 × objective.

Tomographic images with a size of 2000^3^ voxels were acquired at a voxel size of 1.64 μm, which were then binned (2 × 2 × 2) to obtain images of 1000^3^ voxels with a voxel size of 3.28 μm. The binning was used to decrease the size of the final image. The binning also helped improve the signal-to-noise ratio. A total of 3000 projections with an exposure time of 0.06 s was acquired over 180° rotation for the dry reference scan of the sample (without any fluids). For time-resolved imaging during drainage and imbibition, we collected 800 projections with an exposure time of 0.02 s for each tomographic image. The total acquisition time for each time-resolved tomographic image was 24 s (16 s for acquisition and 8 s for triggering). The real time-step between each image was 38 s. This also included 14 s for repositioning the rotation stage to its initial position and transferring the data to a storage disk. During the complete drainage and imbibition processes, we acquired a total of 496 and 416 tomographic images respectively. The images were reconstructed using a filtered back-projection algorithm^35^. A cylindrical mask equivalent to the diameter of the rock sample was applied on the reconstructed data to remove unwanted regions including the Viton sleeve, followed by its conversion from 32-bit to 16-bit to reduce the size, using ImageJ software (https://imagej.net). Hereafter, all the image-processing steps were performed using Avizo-9 software (https://www.fei.com/software/amira-avizo/).

**Figure 1:**
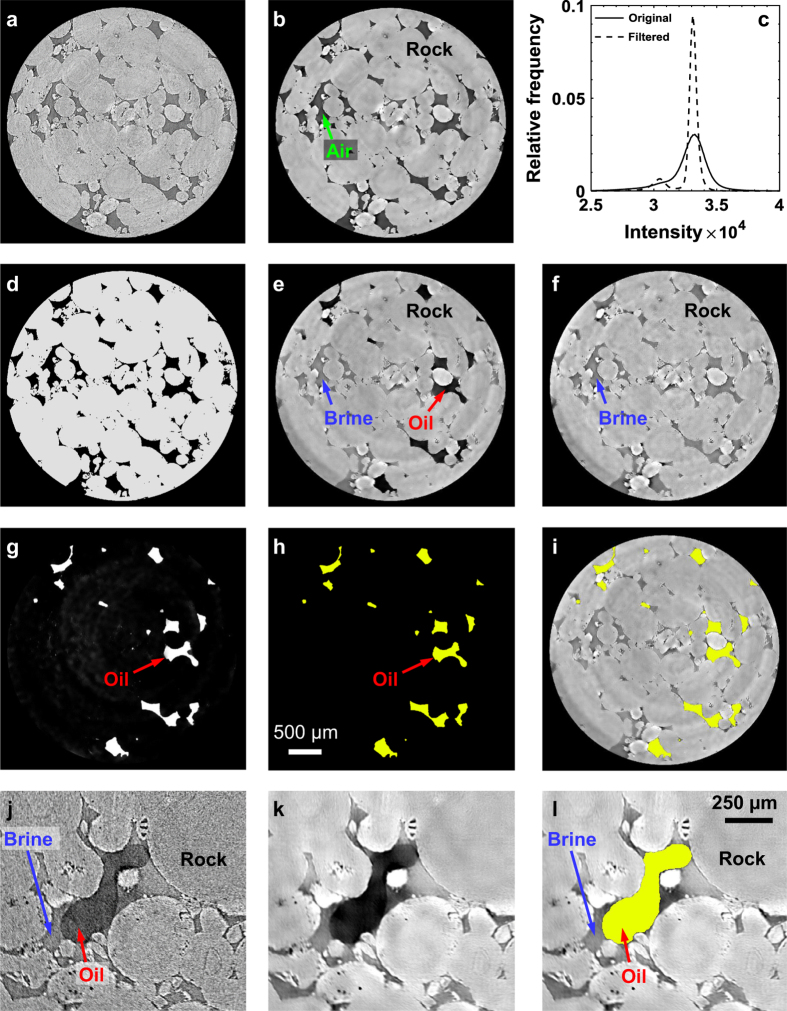
Image processing and segmentation. Two-dimensional horizontal cross-sections of the original raw (**a**) and filtered (**b**) dry reference scans. Here, light gray and dark gray represent rock and pore space respectively. (**c**) Histogram showing intensity profiles of the raw and filtered images. (**d**) The filtered image was segmented into two phases (rock and air) using a seeded watershed algorithm based on the gray-scale gradient and gray-scale intensity of each voxel. Here gray and black represent rock and pore (air) respectively. (**e**) Two-dimensional horizontal cross-section of a three-phase filtered tomographic image. (**f**) Two-dimensional horizontal cross-section of the brine saturated tomographic image, which was acquired before injecting oil in the sample. (**g**) The image (**e**) was subtracted from the brine saturated image (**f**) and filtered with a non-local means filter. (**h**) The image was segmented for oil phase using an intensity-based thresholding method. (**i**) Two-dimensional horizontal cross-section showing the segmented oil from (**h**) superimposed on the three-phase filtered tomographic image from (**e**). (**j**) A zoomed-in two-dimensional cross-section of an original raw image showing various phases. (**k**) The same image after the application of a non-local means filter. (**l**) The oil segmented data is superimposed on the filtered image to show the quality of segmentation at the pore-scale.

First, the dry reference scan (Fig. 1a) was filtered with a non-local means edge preserving filter^36,37^ (Fig. 1b) (Data Citation 4). Figure 1c shows the intensity profiles of the original raw and filtered images. Clearly, the filtering process enhances the contrast between air and rock, indicated by two separate peaks in the filtered image. We also compared the interphase signal-to-noise ratio (ISNR) of the original and filtered images. The ISNR was obtained by calculating the means, *m*_*r*_ and *m*_*p*_, and standard deviation, σ_*r*_ and σ_*p*_, of the gray-scale values within the rock and the pore space^38^.

$$Interphase\;signal-to-noise\;ratio=\frac{\left|m_{r}-m_{p}\right|}{\frac{1}{2}\left(\sigma_{r}+\sigma_{p}\right)}$$

The value of the ISNR increased from 2.8 (for the original image) to 8.8 (for the filtered image), indicating a significant enhancement of signal (or reduction of noise) in the image.

The filtered dry reference scan was then segmented (binarized) into two phases (pore and solid) with a seeded watershed algorithm based on the gray-scale gradient and gray-scale intensity of each voxel^39^ (Fig. 1d) (Data Citation 4). Here, the rock and pore space are shown in gray and black respectively. All the filtered time-series images were then registered to the filtered dry reference scan using normalized mutual information and resampled onto the same voxel grid as the dry reference scan. Each time-series filtered tomographic image containing three phases (Fig. 1e) (Data Citation 4) was then subtracted from the brine-saturated image (Fig. 1f) (Data Citation 4). The image subtraction not only helped in enhancing the contrast between oil and the other phases, but also canceled out the effect of phase-contrast represented by dark spots in the images (e.g., Fig. 1f). These subtracted images were then again filtered using a non-local means filter to increase the signal-to-noise ratio (Fig. 1g). The filtered images were segmented for the oil phase using intensity-based thresholding (Fig. 1h) (Data Citation 4). Figure 1i shows the segmented oil superimposed on the three-phase filtered image (Fig. 1e), which shows the effective segmentation based on the subtracted images. The quality of non-local means filtering and segmentation was also inspected at the pore scale. Figure 1j shows a zoomed-in image of the original raw scan containing three phases. Figure 1k shows the same location after applying non-local means filter, indicating that the filtering improves the signal-to-noise ratio significantly while keeping the phase boundaries preserved. Figure 1l shows the segmented oil superimposed on the filtered image (Fig. 1k), which indicates that segmentation process based on subtracted images captured the oil phase boundary effectively. The oil-segmented datasets were used by Singh, *et al.*^22^ for curvature and capillary pressure analysis during oil snap-off at the pore scale. The three-phase segmented image can be obtained by combining the segmented oil image (Fig. 1h) and the segmented dry reference scan (Fig. 1d).

## Data Records

The data for this manuscript are available on the British Geological Survey (BGS) repository (Data Citation 4), which comprises a dry scan of the rock sample without fluids (unfiltered gray-scale, filtered gray-scale and segmented images), a scan of the rock saturated with brine (unfiltered and filtered gray-scale images), 496 time-series tomographic images of drainage (unfiltered gray-scale, filtered gray-scale and segmented images), and 416 time-series tomographic images of imbibition (unfiltered gray-scale, filtered gray-scale and segmented images). Table 1 shows the details of the tomographic images. It should be noted that the datasets (i) Drainage_time_series_unfiltered, (ii) Drainage_time_series_filtered, (iii) Imbibition_time_series_unfiltered, and (iv) Imbibition_time_series_filtered are not available for downloading from the BGS repository due to their large file sizes. These datasets can be obtained by sending a request to the BGS. Further details are provided in the section Access the dataset (file name: Accessing Data.docx) in Data Citation 4. The rest of the datasets are available for downloading.

Table 2 shows the phase information. Table 3 and Table 4 show the time-step between consecutive time-series tomographic images for drainage and imbibition respectively. In general, images were acquired with a time-step of 38 s. However, the acquisition during imbibition was stopped after scan number 58, 307, 350 and 378 (Table 4) due to failure of the camera control software. Note that the brine injection was not interrupted during rebooting of the camera software. When there was no significant change in oil saturation, we acquired images with a time-step of 950 s (after scan 410 in Table 4) except for the last scan which was acquired with a time-step of 722 s (Table 4).

## Technical Validation

**Figure 2:**
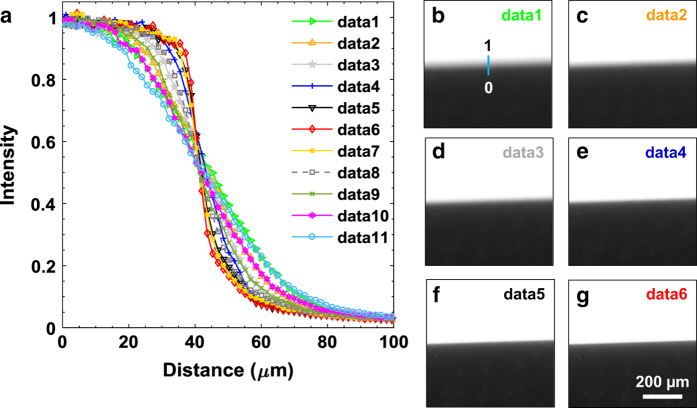
Focusing of the detector. (**a**) Line profiles across the edge of the knife (black in **b**–**g**). The focusing distance between each dataset (data 1 to data 11) is 0.35 mm. (**b**) to (**g**) show the edge of the knife for data 1 to data 6 respectively. The image shifts from blurry to sharp from data 1 (**b**) to data 6 (**g**), and then again to blurry afterwards from data 7 to data 11 (not shown here).

The detector at the beamline I13 (Diamond Light Source) was focused with a high precision 1 mm thick knife edge (JJ X-ray). Multiple images of the knife edge were acquired at different focusing distances, using a PCO Edge camera with a 2 × objective, which were then corrected for dark and flat fields. Figure 2 shows line profiles across the edge of the knife for different focusing distances. Focusing was tuned in steps of 0.05 mm (only larger steps of 0.35 mm are shown in Fig. 2), until a sharp interface was obtained (Fig. 2g corresponding to data 6 in Fig. 2a).

The sample rotation stage was aligned perpendicular to the X-ray beam (pitch alignment) and the rows of the detector within an angular precision of 200 μrad. The axial run-out of the rotation stage is 50 nm and the angular tilt is 0.1 μrad.

## Usage Notes

The tomographic data can be loaded as .raw files using an open source software ‘ImageJ’ (http://imagej.nih.gov/ij). ImageJ has many available plugins for image processing and analysis. For the details of using Imagej for cropping, resizing, adjusting brightness/contrast, readers are referred to Rowe, *et al.*^40^. For three-dimensional visualization, the data can be loaded on an open source software ‘Drishti’ (https://github.com/nci/drishti/wiki).

The data can also be loaded and processed on a commercial software ‘Avizo’ (https://www.fei.com/software/amira-avizo/), which is a powerful tool for data processing, segmentation and analysis. It also has an option of loading and visualizing the complete time-series data. The datasets can also be loaded to any other commercial image processing software.

## Additional information

**How to cite this article**: Singh, K. *et al*. Time-resolved synchrotron X-ray micro-tomography datasets of drainage and imbibition in carbonate rocks. *Sci. Data*. 5:180265 doi: 10.1038/sdata.2018.265 (2018).

**Publisher’s note**: Springer Nature remains neutral with regard to jurisdictional claims in published maps and institutional affiliations.

## Supplementary Material



## Figures and Tables

**Table 1:** Data files and parameters.

File name	No. of scans	Image size (XYZ)	Voxel size (μm)	Image type
Dry_reference_scan_unfiltered	1	1189 × 1163 × 1000	3.28	16-bit gray-scale
Dry_reference_scan_filtered	1	1189 × 1163 × 1000	3.28	16-bit gray-scale
Dry_reference_scan_segmented	1	1189 × 1163 × 1000	3.28	8-bit binary
Brine_saturated_unfiltered	1	1189 × 1163 × 1000	3.28	16-bit gray-scale
Brine_saturated_filtered	1	1189 × 1163 × 1000	3.28	16-bit gray-scale
Drainage_time_series_unfiltered	496	1189 × 1163 × 1000	3.28	16-bit gray-scale
Drainage_time_series_filtered	496	1189 × 1163 × 1000	3.28	16-bit gray-scale
Drainage_time_series_oil_seged*	496	1189 × 1163 × 1000	3.28	8-bit binary
Imbibition_time_series_unfiltered	416	1189 × 1163 × 1000	3.28	16-bit gray-scale
Imbibition_time_series_filtered	416	1189 × 1163 × 1000	3.28	16-bit gray-scale
Imbibition_time_series_oil_seged*	416	1189 × 1163 × 1000	3.28	8-bit binary
Outer_mask_file	1	1189 × 1163 × 1000	3.28	8-bit binary

**Table 2:** Phase information.

File name	Phase information
Dry_reference_scan_unfiltered	Pore space – dark-gray; and rock – light-gray.
Dry_reference_scan_filtered	Pore space – dark-gray; and rock – light-gray.
Dry_reference_scan_segmented	Pore space – 0; and rock – 1.
Brine_saturated_unfiltered	Brine – dark-gray; and rock – light-gray.
Brine_saturated_filtered	Brine – dark-gray; and rock – light-gray.
Drainage_time_series_unfiltered	Oil – black; brine – dark-gray; and rock – light-gray.
Drainage_time_series_filtered	Oil – black; brine – dark-gray; and rock – light-gray.
Drainage_time_series_oil_seged*	Oil – 1; and rest – 0.
Imbibition_time_series_unfiltered	Oil – black; brine – dark-gray; and rock – light-gray.
Imbibition_time_series_filtered	Oil – black; brine – dark-gray; and rock – light-gray.
Imbibition_time_series_oil_seged*	Oil – 1; and rest – 0.
Outer_mask_file	Outer mask – 0; and rest – 1.
*In Table 1 and Table 2, ‘seged’ refers to ‘segmented’.	

**Table 3:** Time steps between consecutive tomographic images for drainage.

Drainage time-series data	Time step (s)
001 to 496	38

**Table 4:** Time steps between consecutive tomographic images for imbibition.

Imbibition time-series data	Time step (s)
001 to 058	38
058 to 059	76
059 to 307	38
307 to 308	76
308 to 350	38
350 to 351	720
351 to 378	38
378 to 379	3120
379 to 410	38
410 to 415	950
415 to 416	722
